# Wide Variation in Virulence and Genetic Diversity of Binucleate *Rhizoctonia* Isolates Associated with Root Rot of Strawberry in Western Australia

**DOI:** 10.1371/journal.pone.0055877

**Published:** 2013-02-06

**Authors:** Xiangling Fang, Patrick M. Finnegan, Martin J. Barbetti

**Affiliations:** 1 School of Plant Biology, Faculty of Science, The University of Western Australia, Crawley, Western Australia, Australia; 2 The University of Western Australia Institute of Agriculture, Faculty of Science, The University of Western Australia, Crawley, Western Australia, Australia; Emory University, United States of America

## Abstract

Strawberry (*Fragaria*×*ananassa*) is one of the most important berry crops in the world. Root rot of strawberry caused by *Rhizoctonia* spp. is a serious threat to commercial strawberry production worldwide. However, there is no information on the genetic diversity and phylogenetic status of *Rhizoctonia* spp. associated with root rot of strawberry in Australia. To address this, a total of 96 *Rhizoctonia* spp. isolates recovered from diseased strawberry plants in Western Australia were characterized for their nuclear condition, virulence, genetic diversity and phylogenetic status. All the isolates were found to be binucleate *Rhizoctonia* (BNR). Sixty-five of the 96 BNR isolates were pathogenic on strawberry, but with wide variation in virulence, with 25 isolates having high virulence. Sequence analysis of the internal transcribed spacers of the ribosomal DNA separated the 65 pathogenic BNR isolates into six distinct clades. The sequence analysis also separated reference BNR isolates from strawberry or other crops across the world into clades that correspond to their respective anastomosis group (AG). Some of the pathogenic BNR isolates from this study were embedded in the clades for AG-A, AG-K and AG-I, while other isolates formed clades that were sister to the clades specific for AG-G, AG-B, AG-I and AG-C. There was no significant association between genetic diversity and virulence of these BNR isolates. This study demonstrates that pathogenic BNR isolates associated with root rot of strawberry in Western Australia have wide genetic diversity, and highlights new genetic groups not previously found to be associated with root rot of strawberry in the world (e.g., AG-B) or in Australia (e.g., AG-G). The wide variation in virulence and genetic diversity identified in this study will be of high value for strawberry breeding programs in selecting, developing and deploying new cultivars with resistance to these multi-genetic groups of BNR.

## Introduction

Strawberry (*Fragaria*×*ananassa*) is one of the most economically important berry crops in the world, with production reaching about 4.4 million tons in 2010 [1], [2]. Root rot of strawberry caused by *Rhizoctonia* spp. is a serious threat to commercial strawberry production worldwide and is associated with severe economic losses, such as have been reported in Japan [3], [4], the USA [5]–[8], Australia [9], [10], South Africa [11], Israel [12] and Italy [13].

*Rhizoctonia* spp. are soil-borne fungal pathogens with a worldwide distribution [14], [15]. They can cause severe damage to a wide range of economically important agricultural and horticultural crops [16]–[18]. Disease caused by *Rhizoctonia* spp. is difficult to manage due to the soil-borne nature and wide host range of *Rhizoctonia* spp. [19], [20]. Management of *Rhizoctonia* root rot on strawberry remains reliant on soil fumigation [13], [21]–[23]. However, the phasing-out of some broad-spectrum pre-planting fumigants due to environmental and health concerns has fostered keen interest in developing alternative non-chemical ways to manage root rot and other soil-borne diseases more effectively and sustainably [23]–[26]. It is now accepted that identifying and deploying resistant cultivars is the most cost effective and environmentally sustainable strategy to control soil-borne disease on strawberry [24]–[27]. However, if novel management strategies, including the breeding of *Rhizoctonia*-resistant cultivars, are to be developed, it is essential to understand the genetic diversity within *Rhizoctonia* and how this diversity pertains to the virulence of isolates.

*Rhizoctonia* spp. have been classified into uninucleate, binucleate and multinucleate *Rhizoctonia* based on the cell nuclear condition [28], [29]. Binucleate *Rhizoctonia* (BNR) have been divided into 21 anastomosis groups (AG-A to AG-U), and multinucleate *Rhizoctonia* (MNR) have been divided into 13 anastomosis groups (AG-1 to AG-13) based on hyphal anastomosis reactions [15], [17], [18], [28]–[30]. Both BNR and MNR have been reported as pathogen groups causing root rot on strawberry. BNR AG-A and AG-G are the two most common groups associated with root rot on strawberry in the world [5], [6], [11]–[13]. BNR AG-I are also found to be associated with root rot on strawberry in the USA and South Africa, while BNR AG-F and AG-K are also found to be associated with root rot on strawberry in Israel [5], [6], [11], [12]. MNR AG-4, AG-5 and AG-6 are also associated with root rot of strawberry in Israel, the USA and South Africa, respectively [5], [11], [12].

The sequence of the internal transcribed spacer (ITS) region of the ribosomal DNA (rDNA) in particular has been found to be very useful for evaluating genetic diversity and characterizing AG groups of *Rhizoctonia* spp. [12], [13], [15], [16], [29], [31], [32]. The ITS sequences lie between the 18S and 5.8S rRNA genes (ITS1) and between the 5.8S and 28S rRNA genes (ITS2). The ITS sequences are present in high copy numbers in all eukaryotic genomes, have a high rate of evolution, and are flanked by highly conserved nucleotide sequences [33]. The grouping of *Rhizoctonia* spp. using molecular analysis based on ITS sequences supports the grouping of *Rhizoctonia* spp. based on classical hyphal anastomosis reactions. Moreover, sequence analysis is rapid, provides higher resolution of relationships and is less technically challenging than grouping of *Rhizoctonia* spp. using classical hyphal anastomosis reactions [12], [20], [29].

*Rhizoctonia* spp. were frequently recovered from diseased plants during outbreaks of root rot on strawberry in Western Australia [10]. However, there is no information on the genetic diversity and phylogenetic status of the *Rhizoctonia* spp. that infect strawberry in Australia. This paper reports studies conducted to (i) characterize the nuclear condition of *Rhizoctonia* spp. isolates recovered from diseased strawberry in Western Australia; (ii) determine their pathogenicity and relative virulence; (iii) define their genetic diversity based on ITS1-5.8S rRNA-ITS2 sequence analysis; (iv) determine if there is an association between genetic diversity and virulence; and (v) determine the phylogenetic relationships of these isolates with those from strawberry and other crops across the world.

## Materials and Methods

### Fungal isolates

A total of 96 *Rhizoctonia* spp. isolates were recovered from discolored root or crown tissues of diseased strawberry plants collected from nine representative commercial strawberry fields in major strawberry production areas in Western Australia. All necessary permits were obtained for the described field studies. The fields were privately owned. The owner of each field permitted us to collect the plant samples. The field studies did not involve endangered or protected species. These isolates were purified by selecting a single hyphal tip (less than 1.5 mm) of each isolate growing on water agar and transferring to fresh water agar to obtain pure cultures, and preserved as lyophilized cultures on colonized millet seeds in glass ampoules. Whenever required, isolates were sub-cultured onto fresh potato dextrose agar (PDA) for subsequent studies.

### Nuclear condition

The numbers of nuclei per hyphal cell in each of the 96 *Rhizoctonia* spp. isolates from strawberry plants were determined according to procedures of Yang et al. [34]. Briefly, a 1 mm^2^ of colonized PDA agar containing mycelia of each isolate from 2 to 3 day-old culture was placed on the middle of a sterilized glass slide coated with a thin layer of PDA. Slides were then maintained on sterile filter papers moistened with sterilized deionized (DI) water in Petri dishes at 25°C for 2 days in the dark. Nuclei were stained with 0.48% (w/v) Hoechst Dye 33258 (Sigma-Aldrich, Australia) in 0.1 M KH_2_PO_4_ buffer (pH 7.8) and in 0.025 M H_3_BO_3_ buffer (pH 10.5). The number of nuclei per cell of each isolate was determined under a fluorescence microscope and photographed (Axioplan 2 microscope, AxioCam Digital photograph system, Carl Zeiss, NSW Australia). The nuclei of 20 cells per isolate were counted to confirm the nuclear status of each isolate.

### Pathogenicity test

Pathogenicity of the 96 *Rhizoctonia* isolates was determined on strawberry seedlings, including eight isolates which had been previously determined [35]. Millet seed-based inoculum of each isolate was prepared using a modified procedure of Fang et al. [35]. Briefly, 200 g millet seed (*Panicum miliaceum*) was soaked in 200 mL DI water in a 1 L flask for 12 h, excess water drained and subsequently autoclaved at 121°C for 20 min on three consecutive days. Six 3 mm-diameter disks from margins of one-week-old colonies of each isolate growing on PDA plates were added to each flask containing sterilized millet seeds. Flasks were shaken every 2 days to ensure uniform colonization and incubated at 22°C for 2 weeks.

A tissue culture system was developed to aseptically produce strawberry seedlings (cv. Camarosa) for this study based on methods described in Fang et al. [25]. Strawberry seedlings were removed from culture tubes after 4 weeks and washed with sterilized DI water. The seedlings were transplanted to plastic pots containing potting mix which were watered daily to free draining with DI water. The potting mix was finely crushed pine bark∶coco peat∶sand at 2.5∶1.0∶1.5 (w/w) and was pasteurized using aerated steam on three consecutive days at 65°C for 90 min prior to use. All pots were maintained in controlled environment rooms (22°C, 16 h photoperiod, 60% relative humidity) and covered for one week with transparent plastic bags to maintain high humidity. After 3 weeks, seedlings were removed from pots and washed with sterilized DI water before using for the pathogenicity tests.

Seedlings were transplanted into plastic pots (9 cm×9 cm) containing pasteurized potting mix infested with millet seed-based inoculum of each isolate at a rate of 0.5% (w/w). Control seedlings for comparison were transplanted into pots containing uninfested potting mix. Uninoculated sterilized millet seed was deliberately not used as a control comparison as it is known to have a ‘baiting-out’ effect on other potential pathogens in the soil, especially *Pythium* spp. when introduced uncolonized into soil [36]. There were four seedlings per pot and two replicate pots for each treatment arranged in a randomized block design. All pots were maintained in the same controlled environmental conditions (27°C, 16 h photoperiod, 60% relative humidity). Pots were watered daily to free draining with DI water. Plants were harvested 3 weeks later and the severity of root rot was assessed on a 0 to 5 disease severity scale, where: 0 = root well developed, no discoloration/rot; 1 = <25% root discolored/rotted; 2 = ≥25%, <50% root discolored/rotted; 3 = ≥50%, <75% root discolored/rotted; 4 = ≥75% root discolored/rotted; 5 = all root rotted. Following disease assessment, to fulfill Koch's postulates, pathogen re-isolations were made from pieces of freshly harvested discolored root pieces onto PDA. Re-isolated cultures were examined microscopically to confirm morphological similarities with inoculated isolates and demonstrate that infection was from the inoculated isolates.

This experiment was repeated once. Isolates were considered to be pathogenic if they caused visible root rot symptoms on seedlings in both repeat experiments. Percent disease index (%DI) was calculated for each pathogenic isolate in each experiment by the following formula:
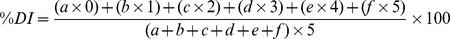
and where a, b, c, d, e and f represent the number of plants with disease scores of 0, 1, 2, 3, 4 and 5, respectively. Data on %DI from the two repeat experiments of each pathogenic isolate were combined and mean %DI was calculated. For all pathogenic isolates, the mean %DI was used to categorize their relatively virulence, where isolates were categorized as high virulence if they showed a mean %DI ≥67, moderate virulence if they showed a mean %DI ≥33 and <67, and low virulence if they showed a mean %DI <33. Only the BNR isolates that were pathogenic on strawberry were subsequently utilized in the molecular studies.

### DNA extraction

Genomic DNA of each of the 65 pathogenic BNR isolates was extracted using the method described by Cenis [37]. Briefly, a 1.5 mL tube was filled with 500 µL autoclaved potato dextrose broth. The culture was started by sub-culturing some mycelia of each isolate into the broth and then kept at 25°C for 72 h. The mycelial mat was removed and washed with TE buffer and pelleted by centrifugation for 5 min at 13,000 rpm, and decanting the supernatant. After adding in 300 µL extraction buffer (200 mM Tris-HCl pH 8.5, 250 mM NaCl, 25 mM EDTA and 0.5% (w/v) SDS), the mycelia was crushed with a conical grinder. 150 µL 3 M sodium acetate (pH 5.2) was added, and tubes were placed at −20°C for 10 min. The supernatant was transferred to a fresh tube after centrifugation for 5 min at 13,000 rpm, and an equal volume of isopropanol was added. After at least 5 min at abient temperature, the precipitated DNA was collected by centrifugation for 10 min at 13,000 rpm. The pellet was vacuum dried for several minutes after washing with 70% (w/v) ethanol and re-suspended in 50 µL of TE buffer. The concentration and quality of the extracted DNA was determined using spectrophotometer (NanoDrop 1000 Spectrophotometer, Thermo Scientific).

### PCR amplification and DNA sequencing

PCR amplification of the ITS region of the rDNA, including ITS1, 5.8S rDNA and ITS2 (ITS1-5.8S rDNA-ITS2), for each BNR isolate was performed using primers ITS1 (5′ TCC GTA GGT GAA CCT GCG G 3′) and ITS4 (5′ TCC TCC GCT TAT TGA TAT GC 3′) [38]. Amplification was conducted in 50 µL reaction mixtures containing 2 µL template DNA (about 100 ng), 25 µL 2× master mix (GoTaq Green Master Mix, Promega Corp.), and 1 µL of each primer (10 µM). Amplification was carried out in a thermal cycler (Bio-Rad Laboratories Pty Ltd) with the following program: an initial denaturation at 95°C for 2 min; 35 cycles consisting of denaturation at 95°C for 30 s, annealing at 55°C for 30 s and extension at 72°C for 30 s; a final extension at 72°C for 3 min. A 5 µL aliquot of each PCR product was separated by electrophoresis on 1.5% (w/v) agarose gels stained with ethidium bromide and visualized using a UV transilluminator. The remaining 45 µL of each PCR product were purified (QIAquick PCR Purification Kit, Qiagen Inc., Valencia, CA) according to the manufacture's instructions and directly sequenced in both directions using the same primers used for the PCR amplification (Macrogen Inc., Korea). For all PCR reactions, sterilized ultra-pure water was used as a control in place of fungal genomic DNA to test for contamination of the reagents. PCR amplifications were performed three times. The size of each amplification product was determined by electrophoresis on agarose gels followed by staining with ethidium bromide to ensure the reproducibility of PCR amplification.

### Sequence alignment, analysis and AG determination

The nucleotide sequences generated by sequencing the ITS1-5.8S rDNA-ITS2 region of each isolate in both directions were edited and assembled using BioEdit 7.1 [39] with manual adjustment. Sequences from all these isolates were compared with those in the GenBank nucleotide database provided by the National Center for Biotechnology Information (http://www.ncbi.nlm.nih.gov/) using the BLAST algorithm to determine sequence identity and find the closest match based on maximal percent identity. The ITS1-5.8S rDNA-ITS2 sequences of the 65 BNR isolates generated from this study were deposited in GenBank (Table 1). Additional reference sequences of 55 representative isolates from known AG groups of BNR from strawberry or other crops across the world were retrieved from GenBank database (Table 2). All but two of these reference sequences of the BNR isolates used in this study are associated with publication in a peer-reviewed scientific journal (Table 2). The ITS1-5.8S rDNA-ITS2 sequence of *Athelia rolfsii* FSR-052 (GenBank Accession No. AY684917) was used as the outgroup [12], [20], [29], [31].

**Table 1:** Virulence of the 65 pathogenic binucleate *Rhizoctonia* isolates obtained from diseased strawberry plants in Western Australia that were used in this study.

Isolate	GenBank accession number	Isolated from	Mean %DI±SD^a^	Virulence^b^
WUF-ST-Rhw2	JQ859847	Root	55.9±5.9	Moderate
WUF-ST-Rhw21	JQ859848	Root	45.8±3.2	Moderate
WUF-ST-Rhwf1	JQ859849	Root	27.4±3.7	Low
WUF-ST-Rhwf9	JQ859850	Crown	18.9±5.4	Low
WUF-ST-Rhwf10	JQ859851	Root	20.0±4.3	Low
WUF-ST-Rhwf12	JQ859852	Root	10.9±2.6	Low
WUF-ST-Rhwf13	JQ859853	Root	100	High
WUF-ST-Rhwf14	JQ859854	Crown	84.7±4.9	High
WUF-ST-Rhwf15	JQ859855	Crown	100	High
WUF-ST-Rhwf16	JQ859856	Crown	100	High
WUF-ST-Rhwf17	JQ859857	Root	100	High
WUF-ST-Rhwf18	JQ859858	Root	78.3±4.6	High
WUF-ST-Rhwf19	JQ859859	Root	87.8±6.7	High
WUF-ST-Rhwf20	JQ859860	Root	100	High
WUF-ST-RhT1-1	JQ859861	Root	75.5±4.9	High
WUF-ST-RhT1-2	JQ859862	Root	80.8±6.3	High
WUF-ST-Rhb5	JQ859863	Crown	65.1±4.1	Moderate
WUF-ST-Rhwf4	JQ859864	Root	100	High
WUF-ST-Rhwf5	JQ859865	Root	89.3±5.2	High
WUF-ST-Rhwf24	JQ859866	Root	70.7±3.7	High
WUF-ST-Rhwf25	JQ859867	Root	100	High
WUF-ST-Rhwf26	JQ859868	Root	100	High
WUF-ST-Rhwf27	JQ859869	Root	77.1±8.6	High
WUF-ST-RhT4-8	JQ859870	Root	28.3±2.5	Low
WUF-ST-RhT4-9	JQ859871	Root	11.8±4.0	Low
WUF-ST-RhT4-10	JQ859872	Root	25.7±5.2	Low
WUF-ST-RhT4-12	JQ859873	Root	13.6±2.1	Low
WUF-ST-RhT4-13	JQ859874	Root	31.3±3.2	Low
WUF-ST-RhT4-14	JQ859875	Root	21.6±5.0	Low
WUF-ST-Rhw1	JQ859876	Root	61.0±5.7	Moderate
WUF-ST-Rhw15	JQ859877	Root	100	High
WUF-ST-Rhw16	JQ859878	Root	95.8±6.0	High
WUF-ST-Rhwf2	JQ859879	Root	45.2±5.4	Moderate
WUF-ST-Rhwf3	JQ859880	Root	36.1±1.6	Moderate
WUF-ST-Rhwf6	JQ859881	Root	59.4±3.7	Moderate
WUF-ST-Rhwf7	JQ859882	Root	53.3±2.5	Moderate
WUF-ST-Rhwf8	JQ859883	Root	37.9±3.0	Moderate
WUF-ST-RhT4-2	JQ859884	Crown	27.7±3.3	Low
WUF-ST-RhT4-3	JQ859885	Crown	28.3±4.7	Low
WUF-ST-RhT4-6	JQ859886	Root	58.2±5.9	Moderate
WUF-ST-RhT3-10	JQ859887	Root	51.5±4.9	Moderate
WUF-ST-RhT3-12	JQ859888	Root	34.3±3.8	Moderate
WUF-ST-RhT3-13	JQ859889	Crown	50.2±2.5	Moderate
WUF-ST-RhT3-14	JQ859890	Root	36.9±4.1	Moderate
WUF-ST-RhT3-15	JQ859891	Root	58.5±5.0	Moderate
WUF-ST-RhT3-16	JQ859892	Root	40.1±1.6	Moderate
WUF-ST-RhT3-17	JQ859893	Root	44.6±6.4	Moderate
WUF-ST-RhT4-1	JQ859894	Crown	64.4±3.7	Moderate
WUF-ST-Rhw17	JQ859895	Root	92.8±4.0	High
WUF-ST-Rhw18	JQ859896	Root	100	High
WUF-ST-Rhw19	JQ859897	Root	100	High
WUF-ST-RhT2-6	JQ859898	Root	88.7±5.2	High
WUF-ST-RhT2-7	JQ859899	Root	76.4±5.1	High
WUF-ST-RhT2-8	JQ859900	Root	72.3±4.7	High
WUF-ST-RhT2-9	JQ859901	Root	70.9±1.6	High
WUF-ST-Rhw4	JQ859902	Root	58.7±5.2	Moderate
WUF-ST-Rhw5	JQ859903	Root	44.8±6.0	Moderate
WUF-ST-Rhw6	JQ859904	Root	64.7±3.8	Moderate
WUF-ST-Rhw7	JQ859905	Root	43.4±4.7	Moderate
WUF-ST-Rhw14	JQ859906	Root	53.7±3.8	Moderate
WUF-ST-RhT2-1	JQ859907	Root	31.3±2.5	Low
WUF-ST-RhT2-2	JQ859908	Root	27.5±4.9	Low
WUF-ST-RhT2-5	JQ859909	Root	25.6±3.4	Low
WUF-ST-RhT4-4	JQ859910	Root	22.3±4.7	Low
WUF-ST-RhT4-7	JQ859911	Root	18.7±1.8	Low

a Percent disease index (%DI).

b Isolates were categorized as high virulence if they showed a mean %DI ≥67; moderate virulence if they showed a mean %DI ≥33 and <67; low virulence if they showed a mean %DI <33.

**Table 2:** Details of reference sequences that were retrieved from GenBank database to evaluate the phylogenetic relationships of the 65 pathogenic binucleate *Rhizoctonia* isolates in this study.

Isolate	GenBank accession number	Anastomosis Group	Host plant	Geographic origin	Reference
Am1	DQ102403	AG-A	Strawberry	USA	[12]
Am2	DQ102414	AG-A	Strawberry	USA	[12]
YWK-83	FJ440196	AG-A	Corn	China	Unpublished
SIR-2	AF354091	AG-A	Sweet potato	Japan	[50]
RU56-8	DQ102417	AG-A	Soil	USA	[12]
C-662	AF354092	AG-A	Soil	Japan	[12]
Str3	DQ102421	AG-A	Strawberry	Israel	[12]
Str8	DQ102422	AG-A	Strawberry	Israel	[12]
Str22	DQ102423	AG-A	Strawberry	Israel	[12]
Str23	DQ102424	AG-A	Strawberry	Israel	[12]
Str34	DQ102425	AG-A	Strawberry	Israel	[12]
R2	AY927315	AG-A	Strawberry	Italy	[13]
R38	AY927337	AG-A	Strawberry	Italy	[13]
R55	AY927347	AG-A	Strawberry	Italy	[13]
FCR2604GB	HM623626	AG-K	Unknown	China	Unpublished
Str24	DQ102429	AG-K	Strawberry	Israel	[12]
F523	FJ492158	AG-K	Sugar beet	Idaho, USA	[18]
AC-1	AB122145	AG-K	Onion	Hokkaido, Japan	[51]
56D17	AB286932	AG-K	Sugar beet	Hokkaido, Japan	[31]
SH-10	AB196652	AG-K	Soil	Hokkaido, Japan	[31]
FA59209	AJ242900	AG-K	Unknown	Spain	[31]
Gm1	DQ102395	AG-G	Strawberry	USA	[12]
Gm2	DQ102397	AG-G	Strawberry	USA	[12]
Str16	DQ102401	AG-G	Strawberry	Israel	[12]
Str31	DQ102399	AG-G	Strawberry	Israel	[12]
Str35	DQ102400	AG-G	Strawberry	Israel	[12]
R1	AY738627	AG-G	Strawberry	Italy	[13]
R9	AY927317	AG-G	Strawberry	Italy	[13]
R18	AY927325	AG-G	Strawberry	Italy	[13]
R22	AY927327	AG-G	Strawberry	Italy	[13]
R25	AY927329	AG-G	Strawberry	Italy	[13]
R56	AY927348	AG-G	Strawberry	Italy	[13]
AH-9	AB196646	AG-G	Peanut	Chiba, Japan	[31]
Su-1	AB196647	AG-G	Peanut	Tokyo, Japan	[30]
RU18-1	DQ102430	AG-Bo	Soil	USA	[12]
RU89-1	DQ102431	AG-Bo	Soil	USA	[12]
C-302	AB219143	AG-Bo	Soil	Fukuoka, Japan	[31]
C-484	AB196641	AG-Ba	Rice	Miyagi, Japan	[31]
Scl-2	AB286930	AG-Ba	Rice	Japan	[31]
C-314	AB286931	AG-Ba	Soil	Fukuoka, Japan	[31]
C-350	AB122144	AG-Bb	Rice	Fukuoka, Japan	[31]
C1	AJ000192	AG-Bb	Rice	Malaysia	[52]
C6	AJ000194	AG-Bb	Rice	Japan	[52]
Im1	DQ102443	AG-I	Strawberry	USA	[12]
Im2	DQ102444	AG-I	Strawberry	USA	[12]
Ibs	DQ102442	AG-I	Soil	Israel	[12]
55D21	AB290023	AG-I	Sugar beet	Hokkaido, Japan	[31]
AV-2	AJ419932	AG-I	Mugwort	Japan	[53]
55D25	AB290021	AG-C	Sugar beet	Japan	[31]
Str47	DQ102441	AG-F	Strawberry	Israel	[12]
Str51	DQ102437	AG-F	Strawberry	Israel	[12]
Str56	DQ102438	AG-F	Strawberry	Israel	[12]
Str110	DQ102432	AG-F	Strawberry	Israel	[12]
Str111	DQ102433	AG-F	Strawberry	Israel	[12]
FSR-052	AY684917	*Athelia rolfsii*	Lily	Taiwan	[12], [31]

Alignment of ITS1-5.8S rDNA-ITS2 sequences of the 65 BNR isolates from this study was performed using the multiple alignment program in Clustal W [40]. The alignment was checked by visual inspection and manual adjustment where appropriate. Phylogenetic trees were constructed based on the multiple alignments (Molecular Evolutionary Genetics Analysis software, Version 5, MEGA 5) [41]. Maximum Likelihood (ML) [42], Maximum Parsimony (MP) [43] and Neighbor-Joining (NJ) [44] were used to construct the phylogenetic trees. The ML analysis was conducted using the Tamura-Nei model [42]. The MP analysis was obtained using the Close-Neighbor-Interchange algorithm [43]. NJ analysis was conducted based on the distance matrix produced by the p-distance model [43]. The robustness of each phylogenetic tree was determined by bootstrapping on the basis of 1000 random samples taken from the multiple sequence alignments. All positions containing gaps and missing data were eliminated. Only nodes with bootstrap values ≥70% were considered to be significant. These processes were repeated after incorporating the sequences of the isolates from this study with reference sequences of isolates from known BNR AG groups from strawberry or other crops across the world. The percent sequence identities of the 65 BNR isolates from this study were determined by analyzing the ITS1-5.8S rDNA-ITS2 sequence of each of these isolates by direct pairwise comparisons. The percent sequence identities of the 65 BNR isolates from this study with reference isolates were also determined by direct pairwise comparisons.

Two isolates were randomly selected from within each of the six different genetic groups revealed by sequence analysis of the 65 BNR isolates from this study to determine if they belonged to different AGs by conducting AG tests using the clean-slide technique [45]. Briefly, a 5 mm-diameter disk from the edge of a 3 day-old colony of a BNR isolate was placed on a sterilized glass slide coated with a thin layer of PDA and a mycelial disk from a similarly grown BNR isolate was placed on the slide at a distance of 2 cm from the first disk. Slides were then maintained on sterile filter papers moistened with sterilized DI water in Petri dishes at 25°C for up to 2 days in the dark. The overlapping hyphae were stained with 0.5% (w/v) safranin O (Sigma-Aldrich, Australia) and 3% (w/v) KOH. Hyphal branches of the stained area were examined microscopically to observe the anastomosis reaction. Further, pairs of the two isolates from between the different genetic groups were also similarly tested.

### Statistical analysis

The Mantel test [46] was performed (R, Version 2.15.1, http://www.r-project.org/) with 1000 simulations to determine the association between genetic distance and virulence of the 65 BNR isolates found to be pathogenic on strawberry in this study. The matrix of genetic distance for the 65 BNR isolates was generated by analyzing the ITS1-5.8S rDNA-ITS2 sequence of these isolates using direct pairwise comparisons. The matrix of difference in virulence for the 65 BNR isolates was generated by analyzing the difference in disease index (%DI) of these isolates using direct pairwise comparisons.

## Results

### Nuclear condition and pathogenicity

**Figure 1:**
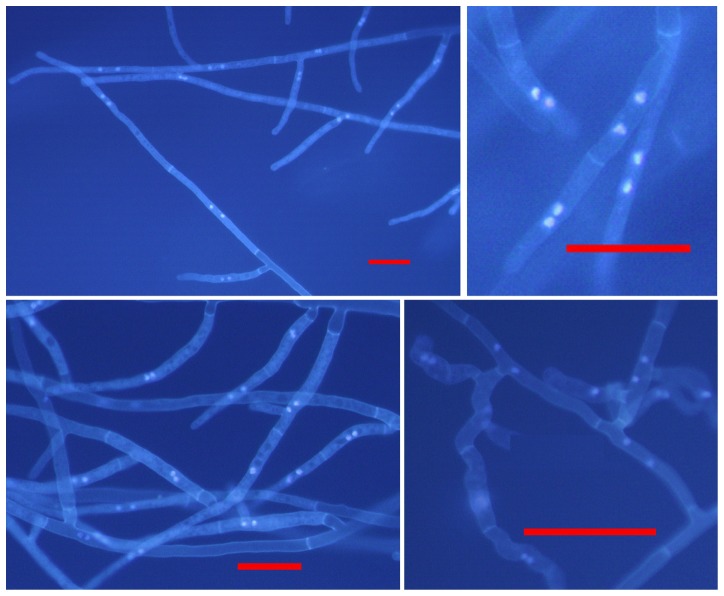
Stained nuclei of representative *Rhizoctonia* isolates from strawberry in this study. Fluorescence micrographs of *Rhizoctonia* isolates stained with 0.48% Hoechst Dye 33258 in 0.1 M KH_2_PO_4_ buffer (pH 7.8). Scale bars: 50 µm.

Nuclear staining of each *Rhizoctonia* isolate showed two nuclei per cell in the hyphae of all the 96 *Rhizoctonia* isolates tested (Fig. 1). This shows that BNR was the predominant *Rhizoctonia* recovered from diseased strawberry in Western Australia. Pathogenicity testing of the 96 BNR isolates showed that 65 isolates were pathogenic on strawberry. Plants in infested soil with these isolates showed root discoloration and/or rot symptoms in replicate experiments. In comparison, control plants in uninfested soil were healthy and did not have any root discoloration and/or rot symptoms. The remaining 31 isolates were non-pathogenic. Plants in infested soil with these isolates did not show any root discoloration and/or rot symptoms in replicate experiments and were similar to the control plants in uninfested soil. Of the 65 pathogenic isolates, 25 had high virulence on strawberry with a mean %DI of greater than 70%, 23 isolates had moderate virulence with a mean %DI of 34 to 65%, and 17 isolates were of low virulence with a mean %DI of 10 to 31% (Table 1).

### Genetic diversity of the 65 pathogenic binucleate *Rhizoctonia* isolates and AG determination

**Figure 2:**
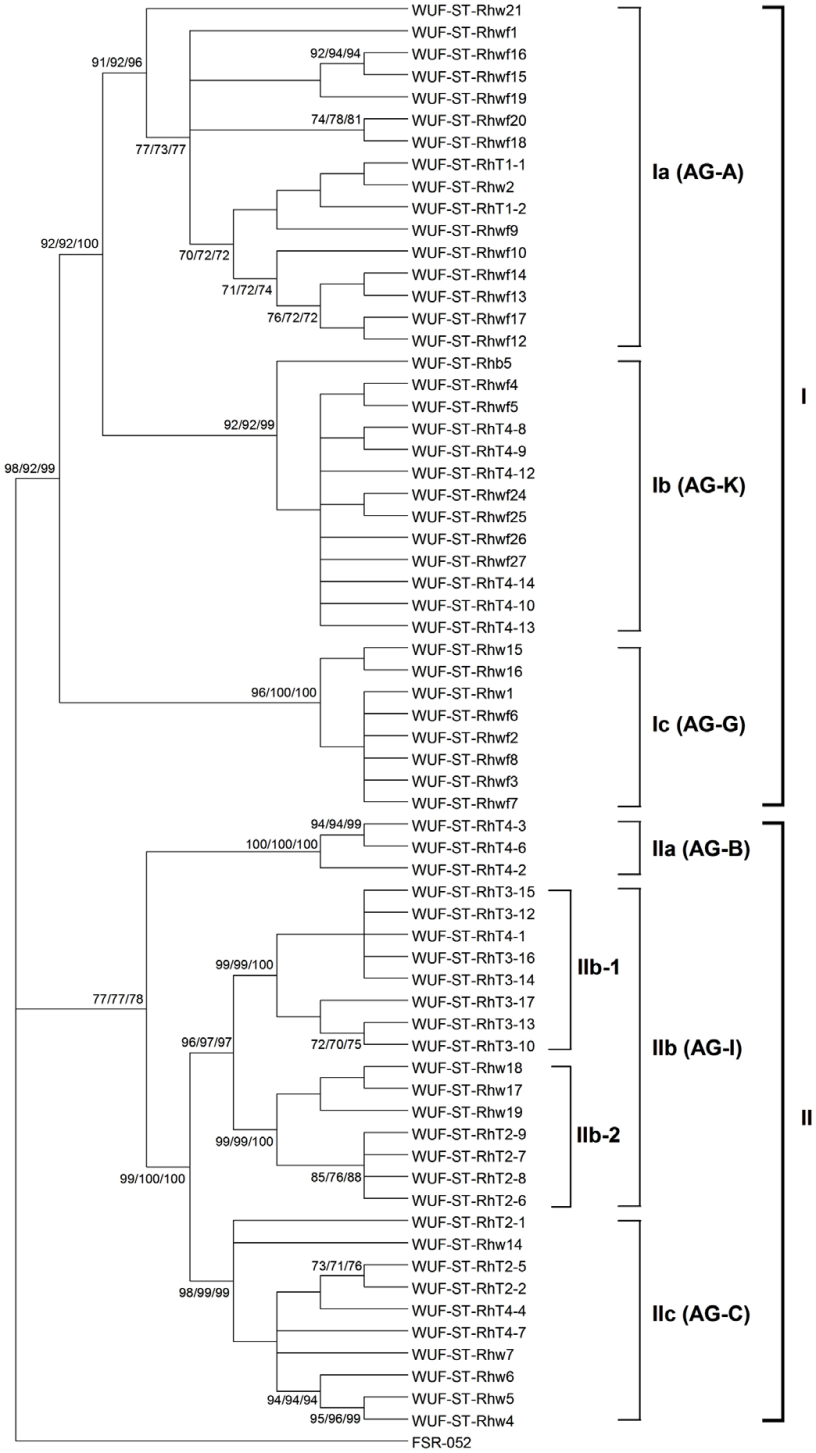
Genetic relationships among the 65 pathogenic binucleate *Rhizoctonia* isolates from strawberry in this study based on the internal transcribed spacer sequences. Trees were constructed using Maximum Likelihood (ML), Maximum Parsimony (MP) and Neighbor-Joining (NJ) analysis. All three methods generated the same tree topology. Tree branches were bootstrapped with 1,000 replications. Numbers at nodes indicate bootstrap values from ML/MP/NJ analysis, respectively. Only bootstrap values ≥70% are shown. The horizontal branch lengths do not represent genetic distance. The tree is rooted with *Athelia rolfsii* FSR-052 (GenBank Accession No. AY684917) as the outgroup. The AG grouping shown on the right of the figure was based on the closest match of the internal transcribed spacer sequences from the isolates in this study with sequences from isolates of known AG in GenBank using the BLAST algorithm.

The ITS1-5.8S rDNA-ITS2 sequence from each of the 65 BNR isolates found to be pathogenic on strawberry (Table 1) was used to determine the genetic diversity of the isolates. Sequence analysis using ML, MP and NJ each found the same tree topology (Fig. 2). The 65 isolates clustered into two distinct clades, with clade I including 37 isolates and clade II including 28 isolates. The 37 isolates in clade I further clustered into three distinct subclades (Ia, Ib and Ic), with bootstrap support by ML, MP and NJ all greater than 90% for each subclade. Clade Ia included 16 isolates, clade Ib included 13 isolates and clade Ic included 8 isolates. The 28 isolates in clade II also clustered into three distinct subclades (IIa, IIb and IIc), with bootstrap support by ML, MP and NJ all greater than 95% for each subclade. Subclade IIa only included three isolates, while subclade IIb included 15 isolates and subclade IIc included 10 isolates. Moreover, subclade IIb was further sub-divided into two subclades (Ib-1 and IIb-2), with bootstrap support by ML, MP and NJ all greater than 98% for each.

Each ITS1-5.8S rDNA-ITS2 sequence of the 65 BNR isolates was compared with sequences in GenBank using the BLAST algorithm to identify the closest match based on maximal identity. None of the ITS1-5.8S rDNA-ITS2 sequences from the 65 BNR isolates were identical with a sequence in the GenBank. In each case, the closest match for every isolate within a clade was found to be in the same AG group (Fig. 2). The ITS1-5.8S rDNA-ITS2 sequences from isolates in clade I were most closely matched to sequences from isolates of AG-A, AG-K and AG-G, while the ITS1-5.8S rDNA-ITS2 sequences from isolates in clade II were most closely matched to sequences from isolates of AG-B, AG-I and AG-C.

The range of the ITS1-5.8S rDNA-ITS2 sequence identity within and between each subclade of the 65 BNR isolates was established by direct pairwise comparisons (Table 3). The sequence identity across all the isolates was 90.1 to 100%. Within the proposed subclades, all the AG-A closely related isolates within subclade Ia had the widest range of sequence identities of 94.4 to 100%, followed by AG-C closely related isolates within subclade IIc which had sequence identities of 96.3 to 100%, and AG-K closely related isolates within subclade Ib which had sequence identities of 98.4 to 100%. All the AG-G closely related isolates within subclade Ic, AG-B closely related isolates within subclade IIa and AG-I closely related isolates within subclade IIb had the sequence identity greater than 99%. Within clades I and II, all isolates had the same sequence identity of 93.0 to 100%. Between the proposed subclades, sequence identities ranged from were 90.1 to 99.3%.

**Table 3:** Ranges of percent sequence identity of the internal transcribed spacer sequences of isolates within and between clades (Fig. 2) of the 65 pathogenic binucleate *Rhizoctonia* isolates in this study.

		Ia	Ib	Ic	IIa	IIb		IIc	Outgroup
						IIb-1	IIb-2		
I**a**		94.4–100							
I**b**		93.0–99.1	98.4–100						
I**c**		93.3–97.2	96.5–97.2	99.8–100					
II**a**		90.5–95.6	94.0–96.0	93.7–94.5	99.3–100				
II**b**	**IIb-1**	90.8–94.7	93.7–95.1	94.4–94.7	95.1–96.0	99.8–100			
	**IIb-2**	91.5–95.2	93.8–95.2	94.9–95.2	95.2–96.1	98.4–98.8	99.8–100		
II**c**		90.1–95.1	92.3–95.4	92.6–95.1	93.0–96.0	96.8–99.3	96.3–98.8	96.3–100	
**Outgroup**		78.3–80.6	79.0–80.1	81.0–81.2	78.7–79.0	80.1–80.3	79.8–79.9	77.6–79.9	100

AG tests were conducted by randomly selecting two isolates from within each of the six different genetic clades (viz. Ia, Ib, Ic, IIa, IIb and IIc) shown in Fig. 2. In each case, the two isolates selected from within the same genetic clade showed an anastomosis reaction, confirming their genetic similarity. In contrast, none of the pairs of the isolates selected from between the different genetic clades showed an anastomosis reaction, confirming their distinct genetic groupings.

### Distribution of the 65 BNR isolates in each genetic clade based on virulence

**Figure 3:**
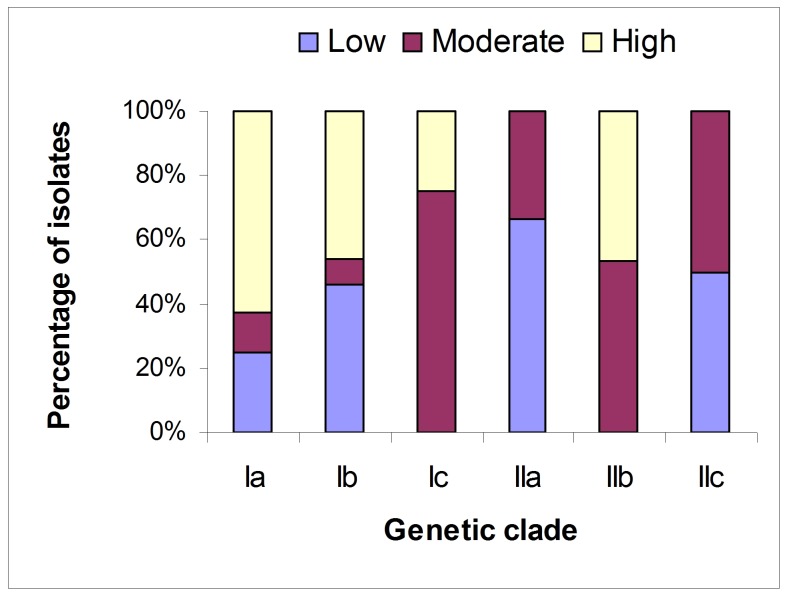
Distribution of the 65 pathogenic binucleate *Rhizoctonia* isolates in each genetic clade based on virulence. Low, moderate and high represent the virulence level of the isolates. Ia, Ib, Ic, IIa, IIb, and IIc represent genetic clades shown in Fig. 2.

There was no significant association between genetic diversity and virulence of the 65 pathogenic BNR isolates (*r* = −0.0974, *p* = 0.6109, Mantel test). None of the observed genetic clades contained isolates from only one category of virulence (Fig. 3). Clades Ia and Ib contained representative isolates from all the three categories of virulence, while the other clades contained representative isolates from two categories of virulence. However, more than half of the isolates with high virulence were distributed in clade Ia, and more than half of the isolates with moderate virulence were distributed in clade Ic. Clades IIb and IIc had about a 50∶50 split between two virulence categories, with moderate/high and moderate/low virulence, respectively. Finally, no clades had only representative isolates from the high/low virulence categories.

### Phylogenetic analysis of the 65 pathogenic binucleate *Rhizoctonia* isolates

The phylogenetic relationships of the 65 pathogenic BNR isolates from this study with the reference BNR isolates from across the world were determined. All the reference isolates included in the analysis were isolates for which the anastomosis group had been determined and the ITS1-5.8S rDNA-ITS2 sequence was available in GenBank. Preliminary analysis found that all reference BNR isolates belonging to AG-A, AG-K and AG-G grouped in clade I, while all reference BNR isolates belonging to AG-B, AG-I and AG-C grouped in clade II. Therefore, isolates in clade I and clade II were analyzed separately.

**Figure 4:**
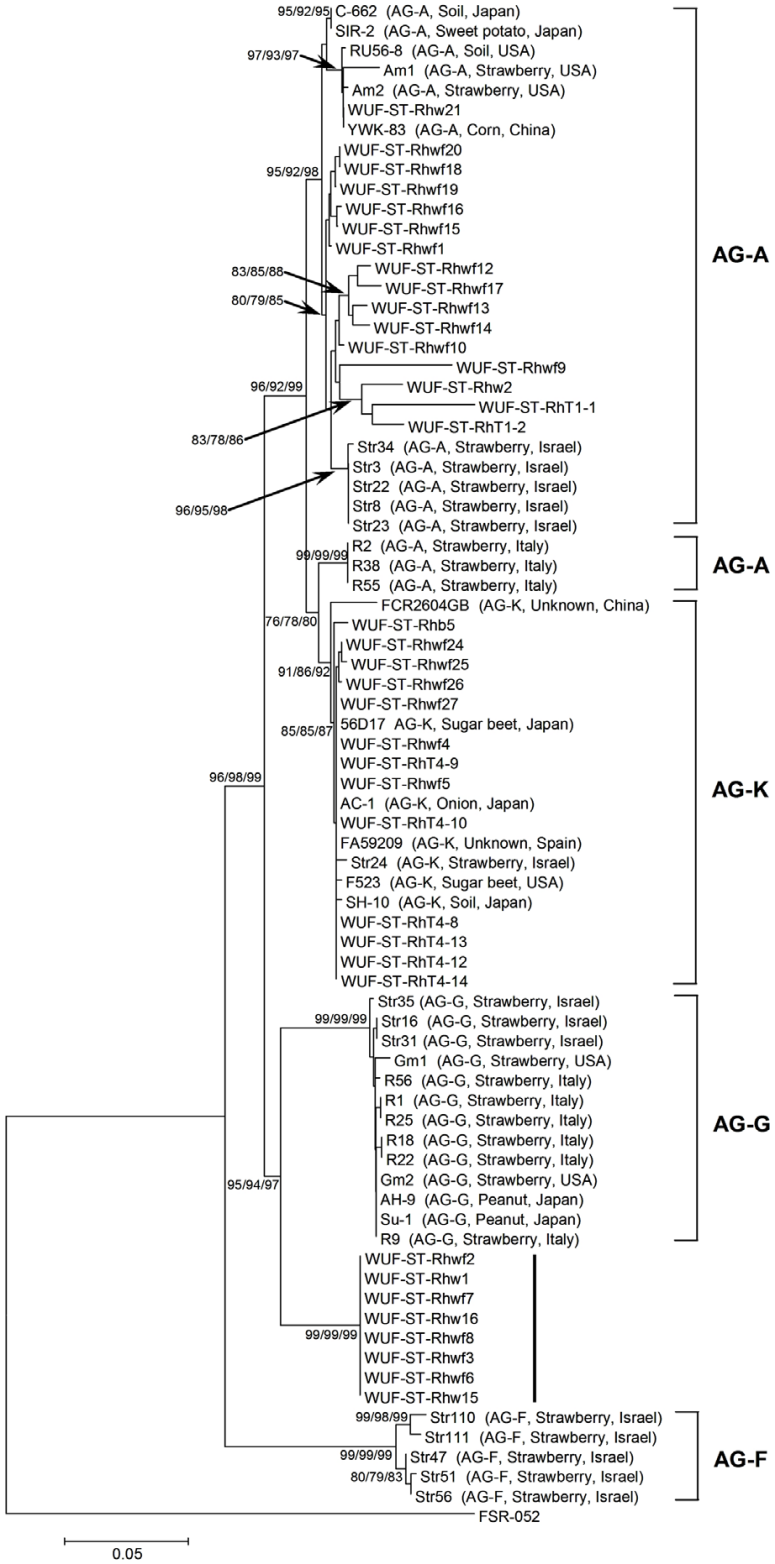
Phylogenetic relationships of the 37 pathogenic binucleate *Rhizoctonia* isolates in clade I (**Fig. 2**) from this study with reference isolates from across the world based on the internal transcribed spacer sequences. Trees were constructed using Maximum Likelihood (ML), Maximum Parsimony (MP) and Neighbor-Joining (NJ) analysis. All three methods generated the same tree topology. Tree branches were bootstrapped with 1,000 replications. Numbers at nodes indicate bootstrap values from ML/MP/NJ analysis, respectively. Only bootstrap values ≥70% are shown. Scale bar represents a genetic distance of 0.05 for horizontal branch lengths. The tree is rooted with isolate *Athelia rolfsii* FSR-052 (GenBank Accession No. AY684917) as the outgroup. The reference isolates are shown as isolate name followed by anastomosis group (AG), host and geographic origin of the isolate in parenthesis. The 37 isolates from this study are shown as isolate name only.

The phylogenetic relationships of the 37 BNR isolates in clade I (Fig. 2) and the reference BNR isolates belonging to AG-A, AG-K, AG-G and AG-F from across the world were determined. Phylogenetic analysis using ML, MP and NJ all found the same tree topology (Fig. 4). The 16 AG-A closely related isolates in subclade Ia clustered into a distinct clade containing nearly all of the reference isolates known to be AG-A. These included isolates from strawberry in the USA and Israel, corn in China, sweet potato in Japan and soil in the USA and Japan. This clade had bootstrap support of 95%, 92% and 98% by ML, MP and NJ, respectively, suggesting that these 16 isolates also belonged to AG-A. The 16 isolates had 94.8 to 99.6% sequence identity with the AG-A reference isolates from strawberry in Israel, and 93.7 to 100% sequence identity with the other reference isolates in this clade. Interestingly, three AG-A reference isolates from strawberry in Italy clustered into a clade that was sister to a clade containing AG-K reference isolates, with bootstrap support of 76%, 78% and 80% by ML, MP and NJ, respectively. The 13 AG-K closely related isolates in subclade Ib clustered into a distinct clade along with all reference isolates known to be AG-K from a variety of crops and geographic origins. This clade had bootstrap support of 91%, 86% and 92% by ML, MP and NJ, respectively, suggesting that these 13 isolates also belonged to AG-K. These 13 isolates had greater than 99.6% sequence identity with the AG-K reference isolates. The eight AG-G closely related isolates in subclade Ic clustered in a distinct clade along with the AG-G reference isolates from strawberry from a variety of geographic origins and peanut in Japan, with bootstrap support of 95%, 94% and 97% by ML, MP and NJ, respectively. However, these eight isolates formed a distinct rooted subclade with a bootstrap support of 99% by each ML, MP and NJ. This clear separation suggested that these eight isolates may represent a new subgroup of AG-G or a new AG group. The sequence identity between these eight isolates and the AG-G reference isolates were 96.0 to 96.7%. None of these BNR isolates in clade I from this study grouped with the AG-F reference isolates from strawberry in Israel.

**Figure 5:**
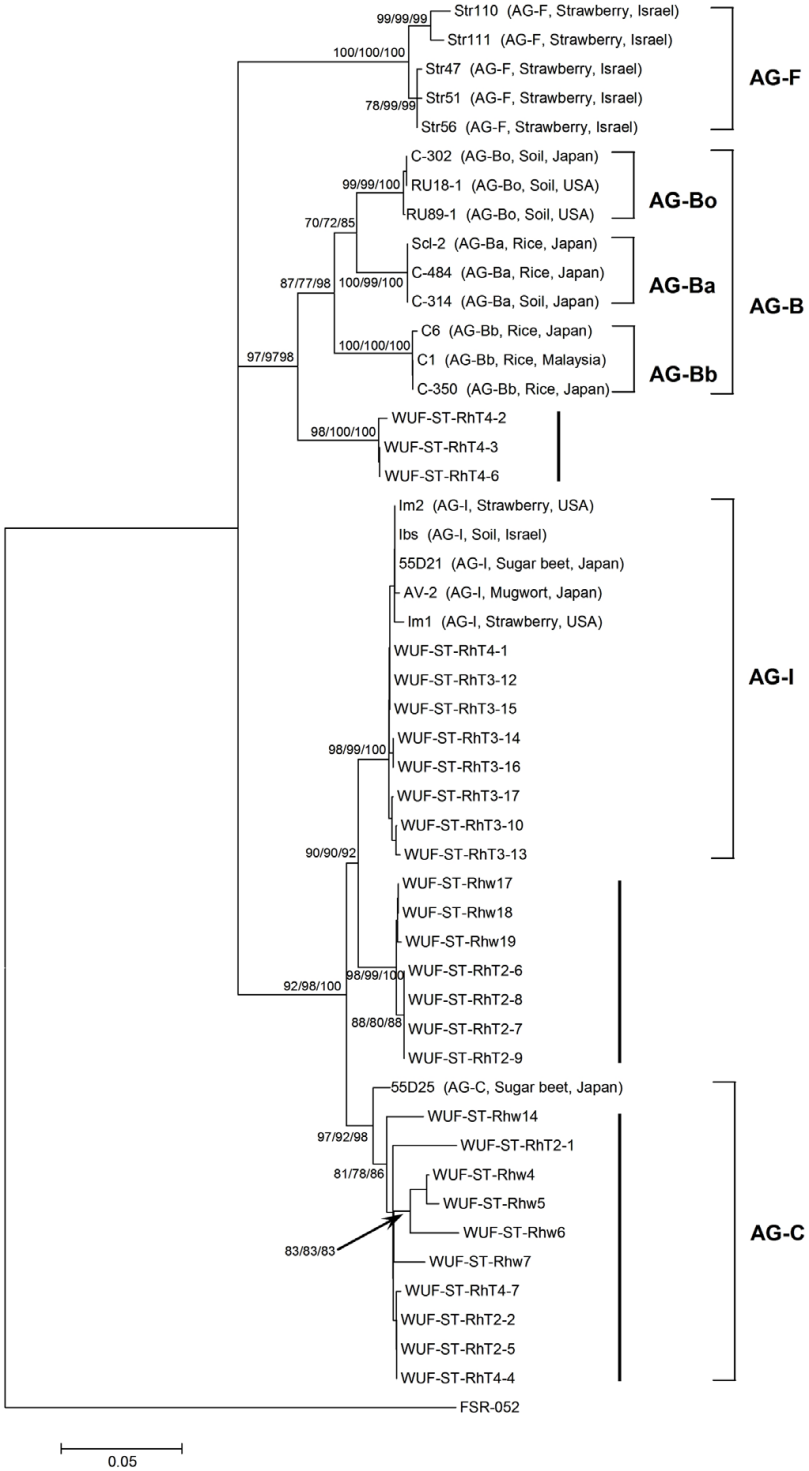
Phylogenetic relationships of the 28 pathogenic binucleate *Rhizoctonia* isolates in clade II (**Fig. 2**) from this study with reference isolates from across the world based on the internal transcribed spacer sequences. Trees were constructed using Maximum Likelihood (ML), Maximum Parsimony (MP) and Neighbor-Joining (NJ) analysis. All three methods generated the same tree topology. Tree branches were bootstrapped with 1,000 replications. Numbers at nodes indicate bootstrap values from ML/MP/NJ analysis, respectively. Only bootstrap values ≥70% are shown. Scale bar represents a genetic distance of 0.05 for horizontal branch lengths. The tree is rooted with isolate *Athelia rolfsii* FSR-052 (GenBank Accession No. AY684917) as the outgroup. The reference isolates are shown as isolate name followed by anastomosis group (AG), host and geographic origin of the isolate in parenthesis. The 28 isolates from this study are shown as isolate name only.

The phylogenetic relationships of the 28 BNR isolates in clade II (Fig. 2) and the reference BNR isolates belonging to AG-B, AG-I, AG-C and AG-F from across the world were determined. Phylogenetic analysis using ML, MP and NJ again all found the same tree topology (Fig. 5). The three AG-B closely related isolates in subclade IIa clustered into a distinct subclade that was sister to a distinct clade containing all the AG-B reference isolates, with the bootstrap support of 97% by both ML and MP, and 98% by NJ. These three isolates had 96.3 to 97.7% sequence identity with the AG-B reference isolates. The AG-B reference isolates clustered into three distinct subclades according to their known subgrouping (AG-Bo, AG-Ba and AG-Bb), with bootstrap support of 99 to 100% by ML, MP and NJ for each. This indicated that the three isolates from this study belonged to a new subgroup of AG-B or a new AG group. The 15 AG-I closely related isolates in subclade IIb clustered into two distinct subclades analogous to IIb-1 and IIb-2 with bootstrap support of 98 to 100% by ML, MP and NJ for each (Fig. 5). The eight isolates in clade IIb-1 clustered into a distinct clade along with all AG-I reference isolates from strawberry, sugar beet, mugwort or soil across a variety of geographic origins, and had greater than 99% sequence identity with the AG-I reference isolates, suggesting that these eight isolates belonged to AG-I. The subclade analogous to subclade IIb-2 contained another seven isolates from this study that may represented a new subgroup of AG-I. These seven isolates had 98.6 to 98.8% sequence identity with the AG-I reference isolates. The 10 AG-C closely related isolates from subclade IIc clustered into a distinct clade along with one AG-C reference isolate from sugar beet in Japan, with bootstrap support of 97%, 92% and 98% by ML, MP and NJ, respectively. This suggested these 10 isolates belong to AG-C. There was 97.3 to 100% sequence identity between these 10 isolates and the AG-C reference isolate. None of the BNR isolates in clade II from this study grouped with the AG-F reference isolates from strawberry in Israel.

## Discussion

All *Rhizoctonia* spp. isolates recovered from diseased strawberry plants in this study were binucleate *Rhizoctonia* (BNR). BNR isolates have been reported as the main isolates recovered from diseased strawberry plants in the USA [5], [6], South Africa [11], Israel [12] and Italy [13]. BNR isolates associated with root rot of strawberry are generally referred to as *R. fragariae* [11], [12], but it is still not clear if BNR only contains one species or rather should be classified into several additional species. While MNR has also been reported recovered from diseased strawberry plants in the USA, South Africa and Israel, this was only at a very low frequency compared with that of BNR [6], [11], [12].

In this study, 65 of the 96 BNR isolates recovered from diseased strawberry were pathogenic on strawberry. It has been reported that BNR isolates recovered from diseased strawberry are also pathogenic on strawberry in other countries, such as in the USA [5], [6], South Africa [11], Israel [12] and Italy [13]. Therefore, this study supports the view that BNR constitutes the major pathogen associated with root rot of strawberry in the world. However, the pathogenic BNR isolates in this study showed a wide variation in virulence, with about 38% of the isolates having high virulence.

It is noteworthy that BNR isolates from this study with the closest match to isolates of the same AG were clustered into the same clade, and the same clade included all the reference isolates from the same AG when reference isolates were included in the analysis. AG determinations confirmed that the BNR isolates from this study that grouped within the same clade belonged to the same AG, while isolates in other clades belonged to different AGs. It has been demonstrated previously that grouping of *Rhizoctonia* isolates by molecular analysis based on the ITS sequences supports the AG grouping of *Rhizoctonia* isolates based on classical hyphal anastomosis reactions [12], [15], [16], [30]. This study confirms that molecular analysis based on the ITS sequences is appropriate for evaluating genetic diversity and characterizing potential AG groups of *Rhizoctonia* isolates.

ITS sequence analysis and AG determination indicated that there were at least six different genetic groups among the pathogenic BNR isolates associated with root rot of strawberry in Western Australia. These groups were inferred to be AG-A, AG-K, AG-G, AG-B, AG-I and AG-C. In addition, the potentials for several new AG groups/subgroups have been highlighted. The two most likely new groups/subgroups are those sister to AG-G and AG-B. The evidence that these may indeed be new groups/subgroups comes from the observation that the split of the new clade from AG-G is more deeply rooted than the split between AG-A and AG-K, with bootstrap support of 99% by each ML, MP and NJ; and that the split of the new clade from AG-B is more deeply rooted than the split between AG-I and AG-C, with bootstrap support of 98% by ML and 100% by both MP and NJ. Another possible new subgroup is that sister to AG-I. The split between this new clade and AG-I is slightly more deeply rooted than the split between AG-I and AG-C, with bootstrap support of 98%, 99% and 100% by ML, MP and NJ, respectively. Finally, the clade of Western Australian isolates that is sister to the clade that includes one AG-C reference isolate may also represent a new AG group/subgroup, although the argument is weaker in this case as there are very few sequences of known AG-C isolates in GenBank available for comparison. These results reveal that there is wide genetic diversity of the pathogenic BNR isolates associated with root rot of strawberry in Western Australia. For example, BNR associated with root rot of strawberry in the USA and South Africa only belong to three genetic groups (viz. AG-A, AG-G and AG-I) [5], [6], [11]. In Italy, only two genetic groups (viz. AG-A and AG-G) are associated with root rot of strawberry [13]. Four genetic groups (viz. AG-A, AG-G, AG-K and AG-F) are associated with root rot of strawberry in Israel [12], but this diversity is still lower than that observed in Western Australia. Therefore, BNR isolates associated with root rot of strawberry in Western Australia are of wide variation in genetic diversity.

This study also showed that there was wide variation in virulence of the pathogenic BNR isolates associated with root rot of strawberry in Western Australia. However, we only used pasteurized soil to test the virulence of the isolates. The virulence of pathogens such as multinucleate *R. solani*, and the activity of antagonistic soil microorganisms on suppressing disease are influenced by soil media [47], [48]. For this reason, it is possible that the observed virulence levels may have been somewhat elevated compared to those in non-pasteurized soil. Despite this consideration, we still did not find an association between genetic diversity and virulence of these BNR isolates. However, this was not surprising as the ITS sequencing in this study was done using generic primers that highlight genetic variation and that were not related to specific virulence or infection-related functions of these BNR isolates.

The lack of association between genetic diversity and virulence of these isolates makes it much more complex in terms of trying to define the distributions of different BNR groups and populations of varying virulence, increasing the challenge of effectively managing root rot of strawberry in Western Australia, such as in selecting, developing and deploying new cultivars with resistance to these different multi-genetic groups of BNR. The soil-borne nature of BNR [15], [20], and the ability of BNR to live also as a saprophyte [30], indicate that any selection associated with the infection of a plant host may not quickly reduce and/or eliminate virulent biotypes, especially those with low or moderate virulence. In addition to virulence on strawberry, biotypes with low or moderate virulence may have a selective advantage when growing as saprophytes. The saprophytic ability and virulence on other hosts may contribute to the observed population structure of the BNR isolates associated with root rot of strawberry in Western Australia.

Our study also highlighted a relatively wide range of ITS sequence identity among the 65 BNR isolates from this study, not only between the different groups of the BNR isolates, but also within the groups of AG-A and AG-C. There was 90.1 to 99.1% sequence identity among the different AG groups. There was 94.4 to 100% sequence identity among the BNR isolates within the group of AG-A, and 96.3 to 100% sequence identity among the BNR isolates within the group of AG-C. Some previous attempts have been made to determine the thresholds of ITS sequence identity for differentiating isolates belonging to different AG groups. However, robust thresholds could not be consistently obtained because there were overlaps between the ranges of ITS sequence identities for isolates within groups and the ranges between groups [12], [31]. The relatively wide range of sequence identity within a genetic group indicates the possible existence of subgroups [12], [31].

Of the six different genetic groups inferred from the BNR isolates associated with root rot of strawberry in Western Australia, we believe that this is the first record worldwide for isolates of AG-B being recovered from diseased strawberry plants and also being pathogenic on strawberry. Moreover, these three isolates showed two levels of virulence, with one isolate of moderate virulence and two others of low virulence. This disagrees with a previous report that isolates of AG-B (AG-Bo RU18-1 and RU89-1) from soil in the USA are non-pathogenic on strawberry [12]. For isolates of AG-C, we confirmed an earlier preliminary report of Fang et al. [35] who were the first to report AG-C as a pathogen of strawberry. These AG-C isolates showed two levels of virulence, with half having moderate virulence and half having low virulence, Isolates of AG-G recovered from diseased strawberry plants represent the first time this group has been shown as a pathogen on strawberry plants in Australia. Two of these AG-G isolates had high virulence, while the remainder had moderate virulence. AG-G has been reported as one common group associated with root rot of strawberry elsewhere in the world [5], [6], [11]–[13]. The majority of the AG-A isolates in Western Australia were of high virulence on strawberry; BNR AG-A is another common group associated with root rot of strawberry elsewhere in the world [5], [6], [11]–[13]. AG-I has been reported associated with root rot of strawberry in the USA and South Africa [5], [6], [11], and also was associated with root rot of strawberry in Western Australia. Previously, only a single isolate of AG-K had been found associated with root rot of strawberry in Israel [12], but in our study, AG-K was one of the main groups associated with root rot of strawberry in Western Australia. However, none of the BNR isolates associated with root rot of strawberry in Western Australia belong to AG-F, a group which only has previously been reported associated with root rot of strawberry in Israel [12].

While currently available ITS sequences from BNR isolates remain quite limited in public databases, increasing the number of sequences in the future should allow even more groups or subgroups of BNR isolates to be defined, and also will be useful for further studies on the genetic diversity of BNR isolates associated with root rot of strawberry worldwide. In particular, additional sequences from other DNA loci such as the beta-tubulin gene and the 28S large subunit of rDNA region, that have been used to determine the genetic diversity and phylogeny of multinucleate *R. solani* [49], could be useful in future not only to further confirm the genetic diversity of BNR isolates associated with root disease of strawberry. but perhaps more importantly in helping to further confirm and delineate the new AG groups or subgroups of BNR as highlighted in this study. Together, this will make it possible not only to improve the current knowledge about BNR as an agent of strawberry root rot worldwide, but also will help the development of novel management strategies on such a serious disease.

In conclusion, this study provides, for the first time, detailed information on the virulence, genetic diversity and phylogenetic status of BNR isolates associated with root rot of strawberry in Western Australia. ITS sequence analysis and AG determination indicated the existence of at least six genetically distinct groups. In addition, the potential for several new AG groups or subgroups of AG-B, AG-G, AG-I and AG-C have been highlighted. This study demonstrates that BNR isolates associated with root rot of strawberry in Western Australia are of wide genetic diversity, with the existence of some potential new genetic groups highlighted for the first time in the world. The wide variation in virulence and genetic diversity, and the lack of an association between genetic diversity and virulence of these BNR isolates associated with root rot of strawberry in Western Australia, together make this disease very challenging to manage. Despite this, the wide variation in virulence and genetic diversity identified in this study will be of high value for strawberry breeding programs in selecting, developing and deploying new cultivars with resistance to these multi-genetic groups of BNR.
